# Re-evaluation of a microbiological acceptable daily intake for tylosin based on its impact on human intestinal microflora

**DOI:** 10.1007/s43188-023-00179-z

**Published:** 2023-08-02

**Authors:** Min Ji Kim, Ji Young Kim, Jang Duck Choi, Guiim Moon

**Affiliations:** Pesticide and Veterinary Drug Residues Division, National Institute of Food and Drug Safety Evaluation, Ministry of Food and Drug Safety, 187 Osongsaengmyeong 2-ro, Osong, Cheongju, Chungcheongbuk-do 28159 Republic of Korea

**Keywords:** Risk assessment, Tylosin, Microbiological acceptable daily intake, Fishes

## Abstract

As veterinary drugs available for fish is very restricted, there is growing trials for repurposing livestock drugs as aquatic animal drugs. Tylosin is one of the most effective antibiotics to treat bacterial infections approved for livestock, and would be used in fish. Hence, we investigated the toxicological and microbiological aspects of tylosin to establish health-based guidance value (HBGV) and maximum residue limit (MRL) in fishes, and reevaluated the microbiological acceptable daily intake (mADI) based on updated relevant data and international guildeline. Lastly, exposure assessment was performed to confirm the appropriateness of MRL. By investigating available microbiologcial studies on tylosin, the microbiological point of departure was determined as 0.308 μg/mL, which was mean 50% minimum inhibitory concentration (MIC_50_), obtained from the Food Safety Committee of Japan (FSCJ) evaluation report. Furthermore, as a factor for the derivation of mADI, the volume of colon content was recently changed to 500 mL in compliance with the International Cooperation on Harmonization of Technical Requirements for Registration of Veterinary Medicinal Products (VICH) guidelines. This was previously defined as the mass of colon content (220 g). We applied correction factor 0.224 to the mean MIC_50_ for tylosin in the equation of mADI, since the drug is transformed to metabolites with reduced activity prior to entering the colon and bound to fecal materials within the colon of human. The mADI was evaluated as 0.01 mg/kg bw/day. Finally, the hazard index, calculated by dividing the estimated chronic dietary exposure by mADI, did not exceed 100%, suggesting that chronic dietary exposure to tylosin residues from veterinary use was unlikely to be a public health concern. Overall, this study contributes significantly in updating HBGV by application of the concept of mADI for the first time in Korea based on the revised microbiological risk assessment guidelines and in providing scientific rationale for the risk management of veterinary drug residues in food.

**Supplementary Information:**

The online version contains supplementary material available at 10.1007/s43188-023-00179-z.

## Introduction

Tylosin is known as a safe veterinary macrolide antibiotic drug used to treat pulmonary disease in chicken and swine and liver abscesses in cattle through feeding or intramuscular (IM) injection. Tylosin has broad-spectrum activity against gram-positive and -negative bacteria and mycoplasma [1, 2]. This compound is originated from soil microorganisms such as *Streptomyces fradiae* [3], and, like other macrolides, it inhibits protein synthesis in bacteria [4].

The drug predominantly consists of tylosin A. The composition of other factors, including desmycosin (factor B), microcin (factor C), and relomycin (factor D), can vary, depending on the manufacturing source. Tylosin A is responsible for most of the microbiological activity, while antibiotic activities of tylosin B, C, and D and dihydrodesmycosin (a metabolite) correspond approximately 50–83%, 70–75%, 30–35%, and 15–31%, respectively, compared to that of tylosin A [3, 5]. Thus, Tylosin with a marker residue as Tylosin A has been set a tolerance level of 0.1 ppm in edible tissues of livestock (i.e., cattle, swine and poultry) in many countries such as European Union, Japan and Korea.

Tylosin is only used in animal husbandry not in human [1]. It was authorized for use in Europe under Regulation (EC) No. 37/2010 in all food-producing species except honeybees. The U. S. Food and Drug Administration (FDA) authorization No. 21 CFR 556.746 permits its use in honey. Similarly, the Japanese Ministry of Food and Drug Safety (MFDS) authorized its use in honey, including royal jelly. 

However, according to a document written by a global network called Health Care Without Harm Europe, antibiotics approved for use in animal husbandry are being repurposed in fish due to drug shortage in aquafarming. In line with the OECD-FAO Agricultural Outlook 2022–2031 [6], global veterinary drug use in aquafarms is anticipated to grow with the increase in fish consumption over the next decades. Several recent studies suggested that tylosin-based antibiotics could be a good candidate to treat bacterial infections in fishes [1, 7]. The pharmaco-kinetics/dynamics profiles of this drug have been explored to determine the optimal treatment regime in fishes [1, 7]. Through a variety of studies on safety and efficacy of tylosin in fish, it was revealed that use of tylosin to fish has lots of advantages, including rapid absorption, low acute toxicity, and no critical adverse effects such as mortality or histopathological changes at appropriate therapeutic doses [1, 7]. Therefore, establishment of maximum residue limit for tylosin in fish is required because of its potential use in aquaculture to increase production by avoiding disease outbreaks.

In addition to establishment of maximum residue limit for tylosinin fish, Health-based guidance value (HBGV) for tylosin is needed to be reevaluated based on its impact on human intestinal microflora. The guideline discusses the general approach and steps to determine a microbiological ADI (mADI) and exhibits the method to calculate of mADI and its equation. Because a value for volume of colon content has been changed from 220 g to 500 mL in updated guideline, mADI of tylosin should be corrected. The other consideration to evaluate mADI of tylosin is to determine microbiological data. In the past, the microbiological data on tylosin as to disruption of colonization barrier was available from JECFA. Recently, the evaluation report of tylosin announced by food safety commision of Japan (FSCJ) has included the microbiological data of tylosin on Japanese's intestinal microbiome. Thus, the consideration of microbiological data obtained from JECFA and FSCJ would be necessary for reevaluation of mADI of tylosin [9].

 Therefore, we will reevaluate the mADI of tylosin following the revised international guidelines [8] considering the up-to-date research results under the agreement of committees with relevant expertise.

## Materials and methods

### Hazard identification

Tylosin toxicity data were collected from evaluation reports issued by international organizations (i.e. Joint FAO/WHO Expert Committee on Food Additives, and Food Safety Commission of Japan) [3, 9].

### Point of departure (POD) determination

The most sensitive endpoint was determined by comparing tylosin toxicological and microbiological data. Additionally, the point of departure (i.e., MIC) determination and the final ADI were based on agreement among the expert committees.

### Exposure assessment

The original Global Estimates of Chronic Dietary Exposure (GECDE) model equation (a) recommended by Joint FAO/WHO Expert Committee on Food Additives (JECFA) [10], was considered for exposure assessment. However, this study used the mGECDE equation (b) for exposure assessment because median residue-level data were not available.

$$\frac{\begin{array}{c}high\, dietary\, expousre \,for \,one\, food \,\left(97.5th \,percentile \,by\, consumers\, \times \,median\, residue\, level\right)\\ +\; mean\, dietary \,exposure \,for\, all \,other\, foods\; \left(average\, consumption\, by \,general\, population\, \times \,median \,residue\, levels\right)\end{array}}{body\, weight\, \left(kg\right)}$$

(a) GECDE equation

$$\frac{\begin{array}{c}high\, dietary \,exposure\, for \,one\, food \; \left(97.5th \,percentile \,by \,consumers \,\times \,proposed \,MRL\right)\\ + \, mean \,dietary \,exposure\,for\, all\, other\, foods\; \left(average\, consumption\, by\, general \,population\,\times \,proposed \,MRL\right)\end{array}}{body \,weight \; \left(kg\right)}$$

(b) mGECDE equation

We estimated the chronic dietary exposure using the 2010–2016 Korea National Health and Nutrition Examination Survey (KNHANES) food consumption data provided by the Korean Disease Control and Prevention Agency (KDCA) and MRL, proposed by the National Fishery Products Quality Management Service (NFQS) and the MFDS. In particular, food consumption data for high consumers were used following the European Food Safety Authority (EFSA) guidance document for exposure assessment [11].

### Risk characterization

The hazard index (HI) was determined by dividing the estimated chronic dietary exposure by the ADI, calculated as follows:

$$\mathrm{Hazard \,Index }\left(\mathrm{\%}\right)=\frac{Human \,daily\, exposure (mg/kg\ bw/day)}{ADI(mg/kg\ bw/day)} \times 100$$

*b.w., body weight

HI ≤ 100%: a hazardous effect is not expected.

HI > 100%: a hazardous effect is expected.

The use of a veterinary drug could be considered as causing no public health concern when the exposure to it is below the threshold (i.e., ADI).

## Results

### Hazard identification

**Fig. 1:**
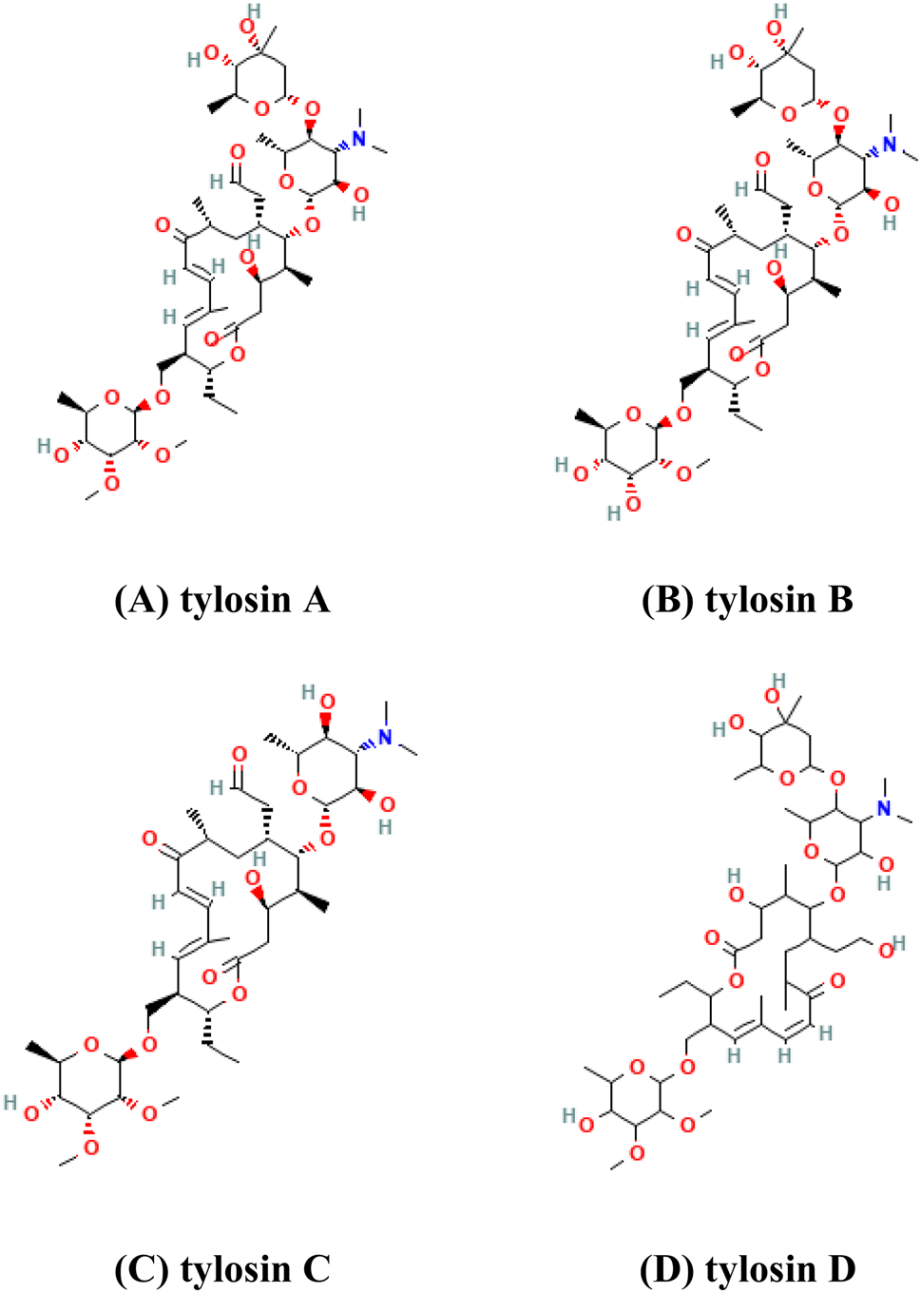
Structures of tylosin factors **a**, **b** (desmycosin), **c** (microcin) and **d** (relomycin)

Various details on tylosin, including its structure, structural identifiers, and physicochemical characteristics, are presented in Fig. 1 and Table 1.

**Table 1:** Physico-chemical characteristics

Generic name	Tylosin
IUPAC Name	[(2R,3R,4E,6E,9R,11R,12S,13S,14R)-12-{[3,6-Dideoxy-4-O-(2,6-dideoxy-3-C-methyl-alpha-L-ribo-hexopyranosyl)-3-(dimethylamino)-beta-D-glucopyranosyl]oxy}-2-ethyl-14-hydroxy-5,9,13-trimethyl-8,16-dioxo-11-(2-oxoethyl)-1-oxacyclohexadeca-4,6-dien-3-yl]methyl 6-deoxy-2,3-di-O-methyl-beta-D-allopyranoside
Molecular formula	C46H77NO17
CAS No	1401-69-0
Molecular weight	916.112 g/mol
Appearance	Off-white to yellow powder
Vapor pressure	0.00 to 8.72 × 10–10 mmHg
Melting point	141℃
Density	1.25 g/cm3
Water solubility	1.74e−4 to 9.81 mol/L
logKow	1.63–3.35

#### Absorption, distribution, metabolism, elimination (ADME)

Based on its pharmacokinetic and pharmacodynamic profiles, tylosin bioavailability is likely to differ among exposure routes and species. Tylosin bioavailability following IM injection in chicken was ranged from 22.5 to 34.0%, while it was ranged from 70 to 95% in pigs, cows, and sheep after oral administration [12–14]. In contrast, the reported oral bioavailability in mink is poor (41%), while the IM-injected bioavailability in olive flounder is high (approximately 87%) [7, 15].

#### Short- and long-term toxicity

Acute toxicity studies performed using various tylosin types, such as base, tartrate, and hydrochloride, routes, and doses indicated that tylosin has low acute toxicity in multiple species [1, 3]. For instance, studies in rats showed an LD_50_ range from 461 to over 6200 mg/kg. Similarly, the LD_50_ range in mice was 321 to over 6200 mg/kg and over 800 mg/kg in dogs. Specific information can be referred to in various unpublished studies such as Anderson et al. (1961), Morten (1988), and Quarles (1983). According to the unpublished paper authored by Richards and Berkman (undated), the LD_50_ in cockerels following a single dose of tylosin phosphate was 3765 mg/kg after oral exposure and 501 mg/kg when injected subcutaneously. Comparative intravenous studies with tylosin A, B, and C in female rats reported respective LD_50_ values of 321, 193, and 189 mg/kg [3].

Several repeated dose studies have been conducted in laboratory animals (rats and dogs), poultry (chickens, turkeys, ducks, and quails), pigs, and cattle. The no-observed-adverse-effect level (NOAEL) for kidney toxicity following oral tylosin administration for two years in dogs was 100 mg/kg bw/day [3]. A similar study by Anderson et al. (1996) unpublished, in which beagles were exposed to up to 400 mg/kg bw/day oral tylosin through capsules for two years, reported no noticeable adverse effects at all concentrations except occasional diarrhea and vomiting at doses of 10–400 mg/kg bw/day. Since diarrhea and vomiting were common symptoms in untreated animals, the NOAEL was considered to be 100 mg/kg bw/day. In rats, the most critical effect was hematological changes at doses greater than 500 mg/kg bw/day; therefore, the EMA concluded that a NOAEL of 50 mg/kg bw/day was appropriate. A one-year study in Wistar rats reported that the 5000-ppm group had increased urinary pH and blood lymphocyte count, and decreased neutrophil count; therefore, the NOAEL was set at 1000 ppm (equivalent to 39 mg/kg bw/day) [9]. Based on the above studies, short- and/or long-term tylosin administration was not considered a cause of critical adverse effects.

#### Reproductive and developmental toxicity

Tylosin was investigated for its teratogenicity and multigenerational reproductive toxicity in rats and mice. Drug-related changes between control and treated groups and critical effects on parents or offspring, including mortality, fertility, and malformation rates, were not observed in various unpublished studies (i.e., Anderson et al., 1996; Broddle et al., 1978; EMEA, 1997; Tsubura et al., undated; Tsuchikawa and Akabori, undated). The NOAELs for teratogenicity and reproductive toxicity could not be determined from these studies. Based on these studies, exposure to tylosin was not expected to cause reproductive or developmental health concerns.

#### Carcinogenicity and genotoxicity

Tylosin was tested for genotoxicity in four in vitro/in vivo assays. Greis (1990) conducted an in vitro chromosome aberration assay in which Chinese hamster ovarian (CHO) cells were treated with tylosin (purity, 99.3%) at 500–1000 µg/mL in DMSO without metabolic activation. The cells were also exposed to 250–750 µg/mL tylosin in DMSO with metabolic activation. The study found no evidence of chromosomal aberrations. Another study used CHO cells for gene mutation assays and found no change in the HGPRT + locus mutation frequency when the tylosin concentration was 100–1500 µg/mL [3]. Greis (1990) also investigated potential gene mutations following treatment with tylosin in mammalian cells. Mouse L5178Y TK + / − cells treated with tylosin (purity, 99.3%) at 10–1000 µg/mL in DMSO showed that tylosin had a weak cytotoxic effect. An in vivo micronucleus assay study performed by Greis (1990) in which ICR mice were administrated up to 5,000 mg/kg bw tylosin (purity, 96%) over 48 h found no evidence of genotoxicity. Therefore, we concluded that tylosin was not a genotoxic compound. Additionally, JECFA reviewed several carcinogenicity studies in rats and found no evidence of carcinogenicity [3]. Overall, tylosin was found to have no genotoxic or carcinogenic effect.

#### Microbiological data

New antibiotics research has highlighted the link between gut microbiota and host health [16]. Furthermore, as the human microbiota is progressively recognized as a critical therapeutic target when using antibiotics [17], the microbiological effects of tylosin have been investigated in several studies. Tylosin residues are known to disrupt the colonization barrier of the gastrointestinal tract in humans because of their antibiotic activities against bacterial strains in the human colonic flora [3]. The most susceptible bacteria were *Bifidobacterium spp.* and *Clostridium spp.,* for which MIC_50_ was 0.062 µg/mL. Conversely, tylosin showed little antibacterial activity against various *Escherichia coli* strains [3]. JECFA committees evaluated tylosin in compliance with the international cooperation on harmonization of technical requirements for registration of veterinary medicinal products (VICH) and clinical and laboratory standard institute (CLSI) guidelines and recognized the need to establish for it a mADI. Although the VICH guideline recommended assessing genera based on their MIC_50_, JECFA committees decided to use the MIC_90_ values for *Bacteroides fragilis*, other *Bacteroides spp., Bifidobacterium spp., Clostridium spp., Enterococcus spp., Eubacterium spp., Fusobacterium spp*., and *Peptostreptococcus spp.* to determine a calculated MIC (MICcalc) as recommended by VICH guideline. The justification for adopting the MIC_90_ was derived from the CLSI guideline. Based on a MIC_90_ of 5.44 µg/mL, the MICcalc was 1.698 µg/mL [3]. A value of 220 g was used for the colon content mass based on the colon content measured in humans. The maximum antibiotic activity available to microorganisms was 64% in human fecal inactivation studies, and the rate its metabolites reach the colon is likely 35% of tylosin in an activity based—pig data as a substitute for human data. Multiplication of these two explained factors yields 0.224, the fraction of an oral dose available to the microorganisms (FA). Therefore, JECFA concluded that an mADI of 0.0–0.03 mg/kg bw/day (rounded-up value as commonly practiced) could be established based on the MIC test and fecal binding data [3]. The formula used for the JECFA evaluation is explained below.

$${\text{mADI }} = \frac{{{\text{MICcalc }}\left( {{1}.{698 }\mu {\text{g}}/{\text{mL}}} \right) \, \times {\text{ mass of colon content }}\left( {{22}0{\text{ g}}} \right)}}{{{\text{FA }}\left( {0.{224}} \right) \, \times {\text{ body weight }}\left( {{6}0{\text{ kg}}} \right)}}$$

In contrast, EMA evaluation of tylosin reported several decades ago established an mADI of 0.006 mg/kg bw/day [5]. The committee for medicinal products for veterinary use (CVPM) in European Union recommended using a specific daily fecal bolus value of 150 mL, geometric mean MIC_50_ for all sensitive genera, and a correction factor (CF) for microbiological risk assessment. The geometric mean MIC_50_ for tylosin was 0.606 µg/mL, calculated from MICs for *Lactobacillus, Bifidobacterium, Clostridium, Bacteroides, Peptostreptococcus, Eubacterium,* and *Enterococcus*. A CF of 2 was used to adjust the inoculum density. An FA of 0.5 was used to account for the nature of the fecal residues because most of the oral dose is excreted through the feces in some species, and data for humans was unavailable. Hence, the formula used by EMA to determine the microbiological risk of tylosin was as follows:

$${\text{mADI }} = \frac{{{\text{geometric mean MIC}}_{{{5}0}} \left( {0.{6}0{6 }\mu {\text{g}}/{\text{mL}}} \right) \, \times {\text{ CF }}\left( {2} \right) \, \times {\text{ daily fecal bolus }}\left( {{15}0{\text{ mL}}} \right)}}{{{\text{FA }}\left( {0.{5}} \right) \, \times {\text{ body weight }}\left( {{6}0{\text{ kg}}} \right)}}$$

However, a recent study by food safety commission of Japan (FSCJ) established an mADI of 0.005 mg/kg bw/day based on the VICH guidelines, with the only difference from the JECFA evaluation being the MICcalc. The FSCJ used a MICcalc value derived from a domestic investigation of the microbiological effects of veterinary antibiotics in 2006. They obtained a MICcalc of 0.308 µg/mL based on MIC_50_ in the same genera following the VICH guidelines [8]. The values for other factors (i.e., FA and colon content mass) remained unchanged. Therefore, an mADI of 0.005 mg/kg bw/day was established in Japan. The FSCJ evaluation formula was as follows:

$${\text{mADI }} = \frac{{{\text{MICcalc }}\left( {0.{3}0{8 }\mu {\text{g}}/{\text{mL}}} \right) \, \times {\text{ mass of colon content }}\left( {{22}0{\text{ g}}} \right)}}{{{\text{FA }}\left( {0.{224}} \right) \, \times {\text{ body weight }}\left( {{6}0{\text{ kg}}} \right)}}$$

### Point of departure (POD) determination

Given the toxicological and microbiological data, the microbiological effects are likely the most sensitive endpoint. Therefore, we reevaluated the data above for mADI. The toxicological and microbiological ADIs of international organizations (i.e., JECFA, EMA, and FSCJ) are summarized in supplementary Tables 1 and 2 to help better understand our conclusions. This study recalculated an mADI following the VICH guideline [8]. The MICcalc data followed the FSCJ approach rather than the JECFA approach, as agreed by the expert committees, for the following reasons. First, in vitro MIC data obtained from Japan could be more reliable than the JECFA data because they are based on enough replicates (preferably ten isolates) and inoculum density (> 10^6^). Second, it is possible that the normal gut microflora in Japanese individuals is comparable to that in Koreans. This opinion could be supported by recent articles on ethnicity-associated differences in gut microflora, although the mechanism remains unclear [18, 19]. However, one of the experts expressed concerns about differences in dietary intake patterns between Koreans and Japanese. Unlike the Japanese, most Koreans not only intake kimchi daily, which food fluently contains the beneficial intestinal bacteria, but also prefer salty and spicy food, and such intake habits could result in microbiome differences [20, 21]. Considering all the above, and with a realistic consideration of the Korean gut microbial communities, using the value obtained from the FSCJ seemed reasonable. These rationales are listed in Table 2. Hence, using the formula below, the most appropriate mADI would be 0.01 mg/kg bw/day.

$${\text{mADI }} = \frac{{{\text{MICcalc }}\left( {0.{3}0{8 }\mu {\text{g}}/{\text{mL}}} \right) \, \times {\text{ volume of colon content }}\left( {{5}00{\text{ mL}}} \right)}}{{{\text{FA }}\left( {0.{224}} \right) \, \times {\text{ body weight }}\left( {{6}0{\text{ kg}}} \right)}}$$

**Table 2:** Korean microbiological ADI and rationales

ADI	0.01 mg/kg bw/day
Study	In vitro microbiological study
Compound	Tylosin
Subjects	Human gut flora from healthy volunteers
Point of departure (MICcalc)	0.308 μg/mL (MIC_50_)
Mass of colon content	500 mL
Fraction of oral dose available to microorganisms	0.224
Body weight	60 kg
Reference	[9]

### Exposure assessment and risk characterization

Chronic dietary exposure to tylosin residues was estimated using the 2010–2016 KNHANES food consumption data and the proposed MRLs. Multiplication of the two factors (MRL and consumption) yields the exposure amount. A detailed explanation of the exposure assessment model was reported by the World Health Organization [22]. A comprehensive explanation of the model was also well-documented as a manual by international organization [10]. The estimated dietary exposure was shown up to 0.2251 mg/person/day (as equivalent to 0.00375 mg/kg bw/day). The hazard index was 37.5%, indicating that the tylosin residues from use are unlikely to cause a public health concern. The exposure assessment results are presented in Table 3.

**Table 3:** Results of estimated chronic dietary exposure and risk (%)

Food	Proposed MRL	MR/TRR ratio*	Corrected TRR value	Intake (kg/day)		Exposure (mg/day)	
				Average	High	Average	High
Fish	0.1	1	0.1	0.0292	0.2191	0.0029	0.0219
Cattle muscle	0.1	0.7	0.1	0.0220	0.2623	0.0031	0.0375
Cattle liver	0.1	0.31	0.3	0.0001	0.1194	< 0.0001	0.0385
Cattle kidney	0.1	0.37	0.3	0.0000	0.0004	< 0.0001	0.0001
Cattle fat	0.1	1	0.1	0.0013	0.0007	0.0001	0.0001
Swine muscle	0.1	1	0.1	0.0471	0.4319	0.0047	0.0432
Swine liver	0.1	0.33	0.3	0.0001	0.0946	< 0.0001	0.0287
Swine kidney	0.1	0.43	0.2	0.0000	0.0000	< 0.0001	< 0.0001
Swine fat	0.1	1	0.1	0.0000	0.0010	< 0.0001	0.0001
Sheep muscle	0.1	1	0.1	0.0000	0.0015	< 0.0001	0.0001
Sheep liver	0.1	1	0.1	0.0000	0.0000	< 0.0001	< 0.0001
Sheep kidney	0.1	1	0.1	0.0000	0.0000	< 0.0001	< 0.0001
Sheep fat	0.1	1	0.1	0.0000	0.0000	< 0.0001	< 0.0001
Goat muscle	0.1	1	0.1	0.0000	0.2184	< 0.0001	0.0218
Goat liver	0.1	1	0.1	0.0000	0.0000	< 0.0001	< 0.0001
Goat kidney	0.1	1	0.1	0.0000	0.0000	< 0.0001	< 0.0001
Goat fat	0.1	1	0.1	0.0000	0.0000	< 0.0001	< 0.0001
Poultry muscle	0.1	1	0.1	0.0270	0.5179	0.0027	0.0518
Poultry liver	0.1	1	0.1	0.0000	0.0000	< 0.0001	< 0.0001
Poultry fat	0.1	1	0.1	0.0000	0.0044	< 0.0001	0.0004
Poultry kidney	0.1	1	0.1	0.0000	0.0000	< 0.0001	< 0.0001
Egg	0.2	0.17	1.2	0.0330	0.1757	0.0388	0.2067
Milk	0.05	1	0.1	0.0963	0.5533	0.0048	0.0277
Sum of exposure (mg/day)						0.2251	
ADI = 0.01 mg/kg bw/day × 60 kg						0.6	
Hazard Index (%)						37.5	

*Marker residue (MR) to total radioactivity residue (TRR) ratios were obtained from EMEA report; Ref. [5]

## Discussion

The present study re-evaluated the Korean ADI of tylosin (0.01 mg/kg b.w./day) based on consultations with expert committees and by performing an exposure assessment using a new dietary exposure model for veterinary drugs. This study is significant as it details mADI derivation for veterinary drugs for the first time in Korea, relying on updated international guidelines.

The modified factor applied for the present study is derived from the investigation on measuring the colon volumes of 75 fasting subjects by using three-dimensional abdominal magnetic resonance imaging (MRI) [23]. The main insight of the investigation has been to find out that the mean value of three regional (ascending, transverse, and descending) colon volumes is about 560 mL. Also, the authors figured out the value for 220 g has been estimated to the lower 95th percentile of colon volumes among those subjects [23]. The value for 220 g had been used as mass of colon content for calculating mADI for long time. For these reasons, World Health Organization (WHO) expert working group concluded that the most appropriate value is considered as 500 mL for colon volume given that mean value for 560 mL except for lower sigmodal colon volume [24].

The limitation of this study was to use old food consumption data in exposure assessment. Hence, there is a need to consider the up-to-date food consumption data when estimating the exposure amounts of residues for reflecting national food consumption pattern. The diet is recognized as a substantial factor for contributing the change of intestinal microflora composition in several research through comparing with western diet and a plenty of fiber diet [25]. Therefore, further investigation of characteristics of intestinal microflora affected by specific food consumption pattern should be warranted. 

### Supplementary Information

Below is the link to the electronic supplementary material.

Supplementary file1 (DOCX 39 KB)
