# Supplementary material for: Re-evaluation of a microbiological acceptable daily intake for tylosin based on its impact on human intestinal microflora
Source: Toxicol Res. 2023 Aug 2;40(1):23–30. doi: 10.1007/s43188-023-00179-z (PMC10786802; doi:10.1007/s43188-023-00179-z)
Supplement: Supplementary file 1 — Supplementary file1 (DOCX 39 KB) [file 43188_2023_179_MOESM1_ESM.docx]

**Supplementary Table 1. Tylosin microbiological ADI reported by international organizations**

| **Regulatory agency** | Study/subjects | Point of departure (MICcalc) | Mass of colon content or daily fecal bolus | FA* | Microbiological ADI | Reference |
| --- | --- | --- | --- | --- | --- | --- |
| **JECFA** | *In vitro* microbiological study  with human gut flora | 1.698 μg/mL  (MIC_90_) | 220 g | 0.224 | 0–0.03 mg/kg bw/day | IPCS & JECFA, 2009  [3] |
| **FSCJ** | *In vitro* microbiological study  with human gut flora | 0.308 μg/mL (MIC_50_) | 220 g | 0.224 | 0–0.005 mg/kg bw/day | FSCJ, 2019 [9] |
| **EMA** | *In vitro* microbiological study  with human gut flora | 0.606 μg/mL (geomean MIC_50_) | 150 mL | 0.5 | 0–0.006 mg/kg bw/day | EMEA, 1997 [5] |
| **APVMA** | - | - | - | - | Not allocated | APVMA, 2022 |
| **FDA** | - | - | - | - | Not allocated | 21 CFR 556.746 |

* Fraction of the oral dose available to the microorganisms

APVMA, 2022 (available at: https://apvma.gov.au/node/26596)

21 CFR 556.746 (available at: https://www.ecfr.gov/current/title-21/chapter-I/subchapter-E/part-556/subpart-B/section-556.74)

**Supplementary Table 2. Tylosin toxicological ADI reported by international organizations**

| **Regulatory agency** | Study | Species | Route/dose | Point of departure/Endpoint | UF | Toxicological ADI | Reference |
| --- | --- | --- | --- | --- | --- | --- | --- |
| **JECFA** | 2-year chronic  toxicity | Beagle dog | PO (capsule), 0–400 mg/kg bw/day | NOAEL 100 mg/kg bw/day based on renal parameters | 100 | 0–1 mg/kg bw/day | IPCS & JECFA, 2009  [3] |
| **FSCJ** | 1-year chronic  toxicity | Wistar rat | PO (diet),  0–10,000 ppm | NOAEL 39 mg/kg bw/day based on changes in lymphocyte and neutrophil counts | 100 | 0–0.39 mg/kg bw/day | FSCJ, 2019 [9] |
| **EMA** | 1-year chronic  toxicity | Rat | PO (diet),  0–1,000 mg/kg bw/day | NOAEL 50 mg/kg bw/day based on changes in lymphocyte and neutrophil counts | 100 | 0–0.5 mg/kg bw/day | EMEA, 1997 [5] |
| **APVMA** | 2-year chronic  toxicity | Rat | PO (diet), - | NOAEL 30 mg/kg bw/day based on pituitary tumors | 100 | 0–0.3 mg/kg bw/day | APVMA, 2022 |
| **FDA** | - | - | - | - | - | Not allocated | 21 CFR 556.746 |

PO, per oral; UF, Uncertainty factor

APVMA, 2022 (available at: https://apvma.gov.au/node/26596)

21 CFR 556.746 (available at: https://www.ecfr.gov/current/title-21/chapter-I/subchapter-E/part-556/subpart-B/section-556.74)
